# Effect of oligomeric lactic acid plasticizer on the mechanical recycling of poly(3-hydroxybutyrate-co-3-hydroxyvalerate)

**DOI:** 10.1007/s11356-023-31758-0

**Published:** 2024-01-12

**Authors:** Ignacio Bernabé, Erika Amarilla, María Ulagares de la Orden, Joaquín Martínez Urreaga, Freddys R. Beltrán

**Affiliations:** 1 Departamento de Ingeniería Química Industrial y Medio Ambiente, ETSI Industriales, Universidad Politécnica de Madrid, 28006 Madrid, Spain; 2 Research Group: “Polímeros, Caracterización y Aplicaciones (POLCA)”, 28006 Madrid, Spain; 3 Departamento Química Orgánica I, Facultad de Óptica y Optometría, Universidad Complutense de Madrid, 28037 Madrid, Spain

**Keywords:** Mechanical recycling, Bioplastic, Biopolyester, Plasticizer, Poly(3-hydroxybutyrate-co-3-hydroxyvalerate), PHBV, Oligomeric lactic acid

## Abstract

Bioplastics such as polyhydroxyalkanoates (PHA) emerge as an interesting alternative to conventional fossil fuel-based plastics and as part of the solution their associated environmental issues. Nevertheless, end-of-life scenarios are still a major concern, especially within a circular economy framework. When feasible, mechanical recycling appears as the best alternative, since it saves raw materials and energy. However, the viability of mechanical recycling can be compromised by the degradation of the plastic during its use and during the recycling process and by the presence of certain additives. Consequently, the main objective of this work is to study the effect of accelerated ageing and mechanical recycling on the structure and properties of poly(3-hydroxybutyrate-co-3-hydroxyvalerate) (PHBV)-based formulations. The obtained results suggest that accelerated ageing and mechanical recycling led only to a slight degradation of the pure PHBV material, along with small variations in the thermal and mechanical properties. However, the plasticized PHBV formulations showed a more severe degradation and increased thermal stability and stiffness, which could be result of the elimination of the plasticizer during the recycling. Overall, mechanical recycling seems to be an interesting valorization strategy for PHBV wastes, although especial attention should be paid to the additives present in the materials.

## Introduction

The utilization of plastics has massively increased in the last decades, reaching the 390 Mt in 2021 (PlasticsEurope 2022). Properties such as their low density, mechanical resistance, and low price have led to plastics being present in wide array of sectors such as packaging, agriculture, automotive industry, construction, and electronics (de Castro et al. 2022; Fernandes et al. 2020). Despite the advantages of these materials, the ecological issues produced by their massive use cannot be ignored. Firstly, more than 95% of plastics are obtained from petroleum, leading to the depletion of a nonrenewable resource. Secondly, the linear economic model is leading to the generation of large amounts of plastic waste which is overwhelming the world’s ability to manage it, thus leading to the accumulation of toxic additives and plastics in the environment (Bonnenfant et al. 2022; Dedieu et al. 2022a, b; Ghaffar et al. 2022). The increasing environmental awareness is leading to the proposal of several approaches for solving the problems derived from the massive use of plastics (Nikiema and Asiedu 2022), such as their substitution, in some applications, for materials with a lower environmental impact. Among these materials are the bioplastics (Mittal et al. 2022), a family of materials which are either biobased, biodegradable, or both. The production of bioplastics has been growing in recent years, with a projected growth from 2.2 Mt in 2022 to 6.3 Mt in 2027 (European Bioplastics 2022).

Among the bioplastics which are gaining a considerable amount of interest are the polyhydroxyalkanoates (PHAs), a family of biodegradable and biobased polyesters which are accumulated intracellularly, by several microorganisms as a carbon and energy storage when exposed to nutrient deficiency and excess carbon. Some authors consider PHAs more environmentally friendly compared to other alternatives such as polylactic acid (PLA), because of the large crop areas, water, and fertilizers needed for its production (Policastro et al. 2021). The most known PHA polymer is the poly(3-hydroxybutyrate) (PHB), which is a homopolyester with high crystallinity, good oxygen and water vapor barrier properties, and improved resistance to UV radiation. However, the high crystallinity of PHB also leads to some drawbacks such as brittleness and narrow processing window (Garcia-Garcia et al. 2022; Tebaldi et al. 2019). Some of these issues can be addressed, at least partially, by using the copolymer poly(3-hydroxybutyrate-co-3-hydroxyvalerate) (PHBV), which shows lower melting and glass transition temperatures than PHB and thus has a wider processing window. Furthermore, it shows lower crystallinity, reduced brittleness, and more degradability the higher the amount of valerate present in the material (Ibrahim et al. 2021).

Despite the improved processability of PHBV, it is still a brittle and rigid polymer. Hence, recently, several researchers have studied the possibility of including plasticizers on PHB and PHBV formulations. Barbosa et al. (Barbosa et al. 2021) reported the plasticizing effect of an oligomeric polyester on a PHBV with 3% HV content. Slongo et al. (Slongo et al. 2018) observed the plasticizing effect of dioctyl phthalate (DOP), epoxidized soybean oil (ESO), and triethyl citrate (TEC) in the thermal and mechanical properties of a PHBV with 3% HV content. In the same line, Kurusu et al. (Kurusu et al. 2015) pointed out that TEC, tributyl citrate (TBC), and tri(ethylene glycol) bis(2-ethylhexanoate) (TEG-EH) increased the ductility of PHB, although special attention to the processing conditions was necessary. Also, Cretois et al. (Crétois et al. 2016) observed that the addition of TEG-EH to PHB led to an increase in the ductility of PHB. García-García et al. (Garcia-Garcia et al. 2016) also reported the plasticizing effect of different chemically modified vegetable oils on PHB, obtaining reduced glass transition temperatures and increased elongation. Parra et al. (Parra et al. 2006) reported a decrease in melting temperatures and an increase in the water vapor permeability after adding poly(ethylene glycol) (PEG) to PHB. A similar behavior was observed by Jost et al. (Jost and Langowski 2015) using six different plasticizers, concisely, propylene glycol, glycerol, TEC, castor oil, ESO, and PEG on a PHBV with a 3% HV content; by Requena et al. (Requena et al. 2016) in a PHBV with 8% HV, by using PEG and two fatty acids; and by Kelly et al. (Kelly et al. 2018) in PHBV (3% HV) and PEG blends. Erceg et al. (Erceg et al. 2005) also observed a decrease in the melting temperature after adding between 10 and 30%wt. of acetyl tributyl citrate (ATBC) to PHB. The plasticizing effect of ATBC, ESO, and PEG was also reported by Panaitescu et al. (Panaitescu et al. 2017) in PHB.

Despite the environmental advantages of using PHAs as substitutes of conventional plastics in some applications, the management of the generated wastes should still be considered in the framework of a circular economy model (Dedieu et al. 2022a, b). In this regard, mechanical recycling still plays a very important role, since it is the most available recycling technology. Furthermore, mechanical recycling allows to not only reduce the amount of wastes, but also reduce the emissions, energy, and consumption of raw materials (Fredi and Dorigato 2021; Kalita and Hakkarainen 2023; Kumar et al. 2023; Niaounakis 2013). There are some studies, such as those conducted by Zaverl et al. (Zaverl et al. 2012) and Dedieu et al. (Dedieu et al. 2022a, Dedieu et al. 2023), which provide useful information on the degradation of PHBV and PHB during melt reprocessing. Furthermore, other works such as those published by Zembouai et al. (Zembouai et al. 2014), Resch-Fauster et al. (Resch-Fauster et al. 2017), Chikh et al. (Chikh et al. 2017), and Shojaeiarani et al. (Shojaeiarani et al. 2019) analyze the effects of thermal reprocessing in PHAs blends with other biopolyesters such as poly(lactic acid) (PLA), poly(butylene succinate) (PBS), and poly(butylene adipate terephthalate) (PBAT). However, there is no data available regarding post-consumer samples and how their service life or other processes present during mechanical recycling of PHBV affect their properties, which could be the key to revalorize previously degraded PHBV fractions.

Consequently, the main aim of this work is to evaluate in detail the effect of simulated service life, washing, and mechanical recycling on the structure and properties of PHBV and plasticized PHBV formulations. To achieve this objective, a commercial grade of PHBV with 1.6% HV content was plasticized by adding oligomeric lactic acid (OLA) and subjected to a melt processing step, followed by an accelerated ageing comprising photochemical, thermal, and hydrolytic degradation. Then, a demanding washing procedure was applied and, finally, a second melt processing step was performed. The obtained materials were characterized by means of solution viscometry, Fourier transform infrared spectroscopy (FTIR), differential scanning calorimetry (DSC), thermogravimetric analysis (TGA), and tensile tests. The obtained results indicate the plasticizing effect of OLA on PHBV, lowering the melting point and increasing the ductility. However, especial attention should be paid during mechanical recycling, since undesirable degradation reactions could take place, thus leading to a recycled material with poor performance.

## Materials and methods

### Materials

A commercial grade of PHBV (ErcrosBio™ PH 016, kindly supplied by Ercros S.A.), with approximately 1.6%wt. 3-hydroxyvalerate (HV) content and a melt mass-flow rate between 5 and 10 g/10 min (2.16 kg at 190 °C) and a density of 1.23 g/ml, was used. Commercial OLA, under the name Glyplast™ OLA2 (kindly supplied by Condensia Química S.A.), was used as plasticizer additive.

### Sample preparation

**Fig. 1:**
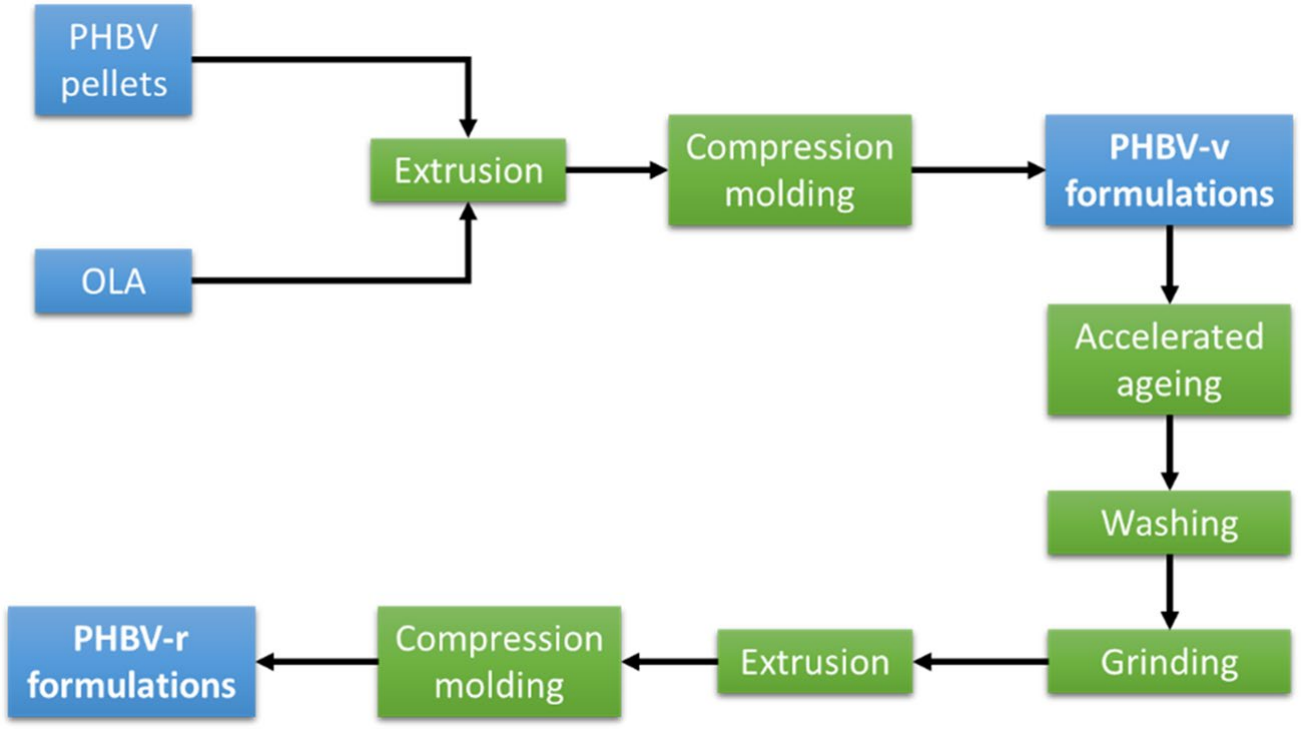
Preparation of samples and mechanical recycling

The analyzed samples were obtained according to the scheme shown in Fig. 1. Pure PHBV pellets and 10, 15, and 20%wt. OLA were melt compounded in a Rondol Microlab twin-screw extruder at 60 RPM. The temperature profile, from hopper to die, was 135–175–185–185–185 °C. Then, the obtained materials were compression molded into films (200 ± 10 µm thickness) using an IQAP-LAP hot-plate press at 185 °C, during 5 min at 14 MPa, yielding the PHBV-v formulations.

The films were then subjected to an accelerated ageing for 240 h, in an Atlas Xenotest 150S chamber equipped with Xenon Arc lamp, under the following conditions: 50% relative humidity, an irradiation level of 1250 W/m^2^, and a black panel temperature of 40 ± 2 °C. The aged samples were washed using a demanding protocol for 15 min at 85 °C, using a NaOH (1%wt.) and Triton X-100 (0.3%wt.) aqueous solution (Beltrán et al. 2018; Chariyachotilert et al. 2012). Lastly, the washed samples were grinded in an IKA A10 analytical mill and reprocessed using the conditions mentioned above. Table 1 summarizes the samples studied in this work.

**Table 1:** Description of materials prepared in this study

Sample	Description
PHBV-v	PHBV material subjected to extrusion molding and compression molding processes
PHBV-10OLA-v	PHBV with 10%wt. OLA subjected to extrusion molding and compression molding processes
PHBV-15OLA-v	PHBV with 15%wt. OLA subjected to extrusion molding and compression molding processes
PHBV-20OLA-v	PHBV with 15%wt. OLA subjected to extrusion molding and compression molding processes
PHBV-r	PHBV-v subjected to accelerated ageing, washed, and reprocessed
PHBV-10OLA-r	PHBV-10OLA-v subjected to accelerated ageing, washed, and reprocessed
PHBV-15OLA-r	PHBV-15OLA-v subjected to accelerated ageing, washed, and reprocessed
PHBV-20OLA-r	PHBV-20OLA-v subjected to accelerated ageing, washed, and reprocessed

### Characterization techniques

Intrinsic viscosity of the samples was measured at (30 ± 0.5) °C, using a type 1 Ubbelohde viscometer and chloroform as solvent. Three different concentrations were measured for each sample. FTIR spectra of all the materials were recorded using a Nicolet iS10 spectrometer equipped with an attenuated total reflectance (ATR) accessory. Sixteen scans, with a 4 cm^−1^ resolution, were recorded for each sample.

Thermal transitions of the materials were studied by means of DSC, in a TA Instruments Q20 calorimeter. Five-milligram samples were weighed in standard aluminum pans and subjected to the following thermal protocol: (i) a first heating scan between − 50 and 200 °C at 10 °C/min; (ii) an isothermal step for 3 min; (iii) a cooling scan between 200 and − 50 °C at 10 °C/min; and (iv) a second heating scan between − 50 and 200 °C at 10 °C/min. All the steps were performed under nitrogen atmosphere (50 ml/min).

Thermal stability was analyzed by means of TGA tests, using a TA Instruments TGA 2050 thermobalance. Ten-milligram samples were heated, in a platinum crucible, between 40 and 800 °C at 10 °C/min. A nitrogen atmosphere (30 ml/min) was used during the experiment.

Tensile tests were carried out using a Shimadzu AGS-X 100 N tensile testing machine, equipped with a 100 N load cell using a crosshead speed of 5 mm/min. Tests were carried out on standard ISO 527–2 type 1BB dumbbell samples.

## Results and discussion

### Intrinsic viscosity

**Fig. 2:**
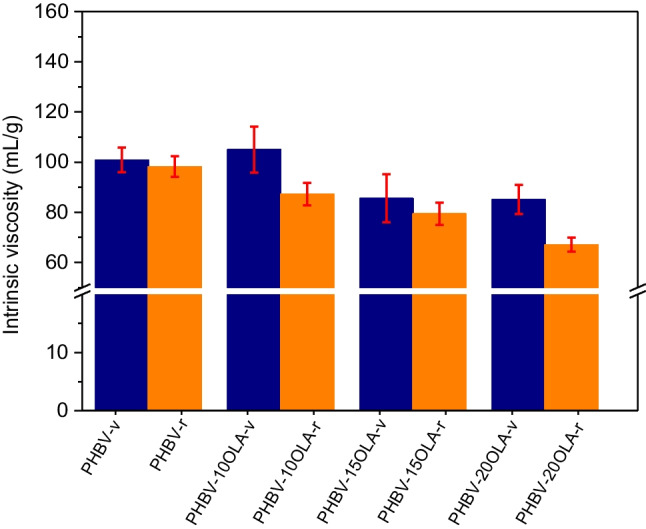
Intrinsic viscosity values of the different materials before and after mechanical recycling

Plastic materials are exposed to several degradation agents during their service life and mechanical recycling, which results in a decrease of the molecular weight and the performance of the samples (Erceg et al. 2005; Pfaendner 2022). PHBV, as many other polyesters, is especially susceptible to hydrolytic and thermal degradation. In the case of hydrolytic degradation, chain scission processes take place mostly toward in ester bonds toward the end of the polymer backbone, resulting in the formation of oligomers which later can be further hydrolyzed, finally yielding monomeric units (Kučera et al. 2019). Regarding thermal degradation, previous studies propose a mechanism based on cis-elimination reactions, which lead to shorter polymer chains and carboxylic acids such as crotonic and 2-pentanoic acid (Nguyen et al. 2002; Vírseda et al. 2023). Finally, another potential degrading agent for PHB and PHBV-based material is light. According to Sadi et al., the main photodegradation mechanism for PHB is free radical-initiated chain scission reactions, which lead to the formation of carbonyl-end species. The free radicals could be originated from a Norrish I reaction, due to the presence of C = O chromophoric groups in the structure of the polymer (Sadi et al. 2010). The combination of all these degradation processes leads to the formation of shorter polymer chains, with reduced intrinsic viscosity; thus, intrinsic viscosity measurements could allow to analyze the effect of the accelerated ageing and mechanical recycling on the different PHBV-OLA samples. Furthermore, intrinsic viscosity is also a crucial parameter in the processing of plastics material; thus, significant variations after service life and recycling could lead to important issues in the quality of the recycled products. Figure 2 shows the intrinsic viscosity values of the different materials before and after the accelerated ageing and mechanical recycling.

It can be seen on Fig. 2 that the addition of OLA leads to a decrease of the intrinsic viscosity of PHVB, especially in the formulations with 15 and 20%wt. This behavior is not surprising, since OLA is a low molecular weight additive. Regarding the effect of mechanical recycling, pure PHBV suffers a slight reduction of the intrinsic viscosity, which evidences slight degradation during the whole process. Similar results have been reported by Zaverl et al. (Zaverl et al. 2012) after one reprocessing cycle, which led to a 2% decrease of the average molecular weight of a PHBV with 14%wt. HV content. In the same line are the results reported by Dedieu et al. (Dedieu et al. 2022a), which found a 1.5% decrease of the average molecular weight after one melt reprocessing cycle in a PHBV sample (3%wt. HV content). The decrease of the molecular weight can be attributed to the unzipping reaction, via a cis-elimination mechanism, in PHBV, as it has been pointed out by several authors (Liu et al. 2009; Shojaeiarani et al. 2019; Xiang et al. 2016).

The behavior of the samples loaded with OLA is similar, although the overall decrease of the intrinsic viscosity is larger in these samples. This result might suggest that OLA is more affected by the mechanical recycling process than the pure PHBV material. The short chains present in OLA could suffer a severe degradation process during the simulated service life and recycling. Furthermore, the degradation of OLA could generate small chains with terminal -COOH and -OH groups that would catalyze the degradation of the polymer matrix during the reprocessing stage (Weng et al. 2010). The catalyzing effect of the terminal carboxyl and hydroxyl groups on the thermo-mechanical degradation of polyesters has already been reported in a previous study with PLA. Beltrán et al. (Beltrán et al. 2018) pointed out that the presence of these terminal groups led to a 20% decrease of the intrinsic viscosity.

### FTIR spectroscopy

**Fig. 3:**
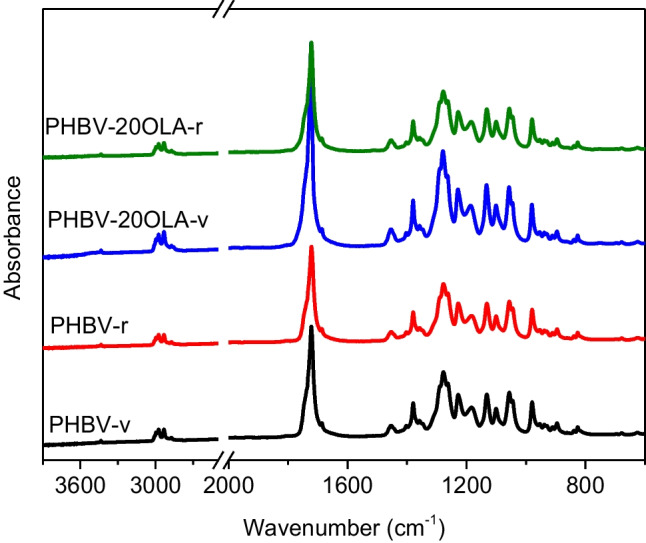
FTIR spectra of pure PHBV and PHBV-20OLA samples before and after recycling

The effect of both the addition of OLA and mechanical recycling on the chemical structure of PHBV was analyzed by means of FTIR-ATR spectroscopy. Figure 3 shows the spectra of pure PHBV and PHBV-20OLA samples before and after recycling. Overall, all the samples showed the characteristic absorption bands of PHBV, which are extensively analyzed in previous studies (Izumi and Temperini 2010; Padermshoke et al. 2004; Pal et al. 2023; Zembouai et al. 2013): stretching of C-H bonds around 2990 cm^−1^, stretching of the C = O group at 1718 cm^−1^ (crystalline regions, with a shoulder at 1740 corresponding to the amorphous phase), stretching of the C-O bonds in the crystalline regions at 1282 cm^−1^, and stretching of the C-O bonds in the amorphous regions at 1188 cm^−1^.

**Fig. 4:**
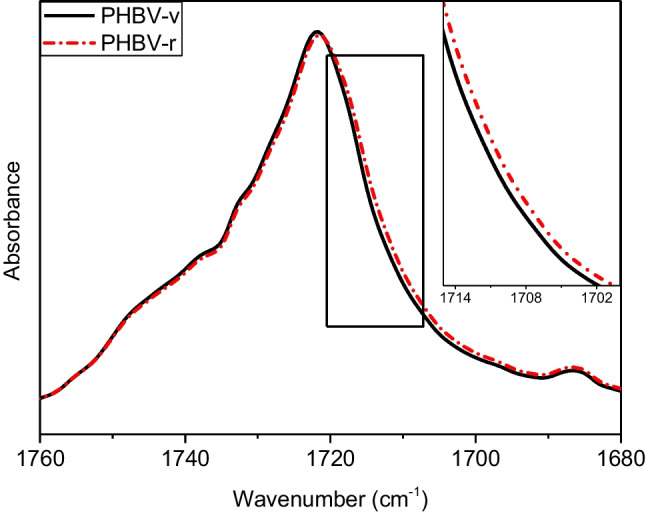
The 1750 cm^−1^ region of the spectra of PHBV before and after recycling

Despite the similarities among the different samples, there are some chemical differences that are worth to be outlined. For instance, there is small displacement toward lower wavenumber of the C = O band after mechanical recycling of pure PHBV. This change can be attributed to the degradation of the polymer during the accelerated ageing and the mechanical recycling process. It is well known that biopolyesters, such as PHBV, are susceptible to thermal and hydrolytic degradation reaction. Such chain scission processes generate -COOH terminated species (Nguyen et al. 2002), which could be responsible of the small displacement of the C = O band toward lower wavenumbers (Izumi and Temperini 2010) (Fig. 4).

**Fig. 5:**
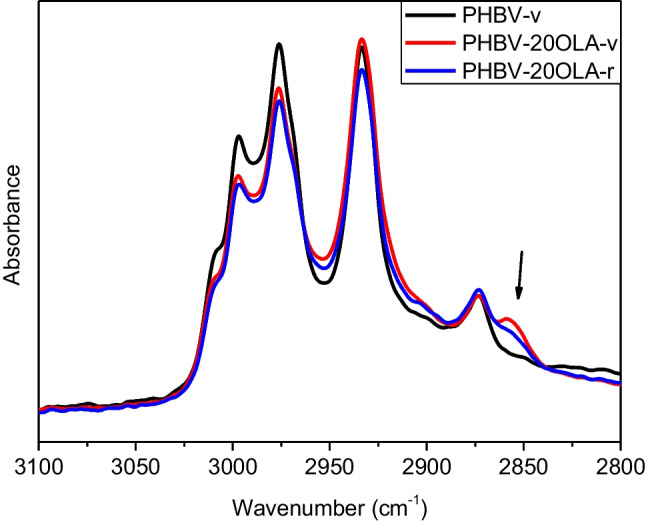
Region around 3000 cm^−1^ of the spectra of PHBV and PHBV-20OLA before and after recycling

The presence of OLA in the samples does not generate important changes in the FTIR spectra, since the most important absorption bands of oligomeric lactic acid are similar to those of PHBV (Jarmelo et al. 2012). Nevertheless, the addition of OLA led to the appearance of small absorption band in the C-H stretching region, concretely around 2855 cm^−1^, as it can be seen in Fig. 5. Furthermore, the absorption band related with the presence of OLA decreases after mechanical recycling, suggesting that part of the OLA is eliminated or degraded during the accelerated ageing and mechanical recycling processes. It is worth to note that plasticizer additives tend to migrate to the surface of the materials, thus making their elimination during the different stages of service life and mechanical recycling more feasible. This result is important, since a lower amount of OLA in the recycled samples would lead to materials with different mechanical and thermal properties.

### Thermal properties

**Fig. 6:**
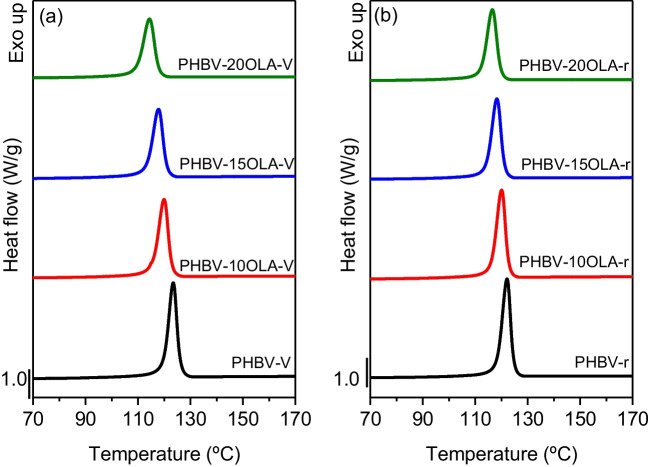
DSC cooling scans of the different PHBV-OLA samples

Thermal properties play a very important role in both processing and service life of plastic materials. The effect of the addition of OLA and mechanical recycling on the thermal transitions and the thermal stability of PHBV was analyzed by means of DSC and TGA tests. Figure 6 and Table 2 summarize the results obtained for the cooling scans.

**Table 2:** DSC cooling scan data for the different PHBV-OLA samples

Sample	*T*_c_ (°C)	Δ*H*_c_ (J/g)
PHBV-v	123.4	96.2
PHBV-10OLA-v	119.9	88.3
PHBV-15OLA-v	117.8	81.4
PHBV-20OLA-v	114.4	75.3
PHBV-r	122.8	92.9
PHBV-10OLA-r	120.0	87.3
PHBV-15OLA-r	118.2	82.9
PHBV-20OLA-r	116.5	76.9

Overall, all the samples show the same behavior, presenting an exothermic peak corresponding to the crystallization of the polymer during cooling. However, there is a decrease of the crystallization temperature with the content of OLA in the materials, indicating that the presence of OLA hinders the crystallization of the PHBV matrix. This result does not agree with the plasticizing effect of OLA in PHBV. Plasticizing molecules are expected to distribute between the PHBV chains, increasing the free volume and the mobility of the molecular chains and thus promoting the formation of crystalline structures (Kurusu et al. 2015; Slongo et al. 2018). However, plasticizers could also have a solvating effect, interfering with the formation of crystalline structures in PHBV (Wypych 2017). Similar results were obtained by Panaitescu et al. (Panaitescu et al. 2017) in PHB samples plasticized with 5%wt. ATBC and ESO, attributing the results to the interaction between the terminal groups of the plasticizer and -COOH groups of PHB. Requena et al. (Requena et al. 2016) also observed a decrease of the crystallization temperature in PHBV samples plasticized with PEG, lauric acid, and stearic acid. Regarding the effect of mechanical recycling, no important differences can be observed in the cooling scans, which suggest that the slight degradation observed by means of intrinsic viscosity does not affect the behavior, during cooling, of the different samples.

**Fig. 7:**
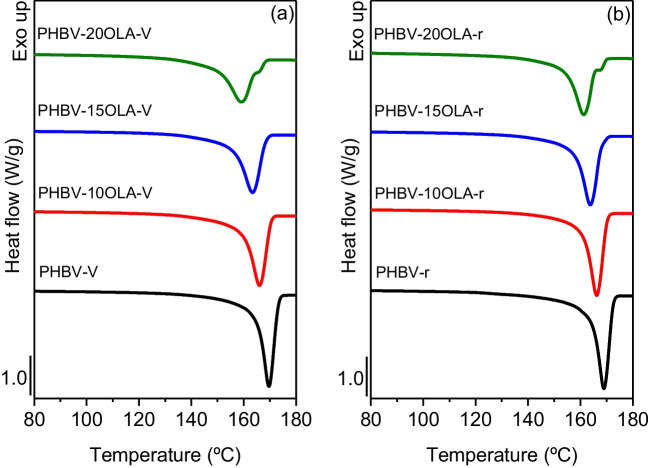
DSC second heating scans of the different PHBV-OLA samples

Figure 7 and Table 3 show the results obtained from the second heating scan of the different materials. All the samples show a similar behavior, with a melting endotherm above 150 °C. It is worth to note that the samples with 20%wt. OLA show a small shoulder at higher temperatures. The presence of a double melting peak in PHBV has been attributed to several causes: melt recrystallization, different lamellar thickness, physical ageing, polymorphism, or different molecular weight species (Panaitescu et al. 2017). Previous studies, such as the one conducted by Janigová et al. (Janigová et al. 2002) and Slongo et al. (Slongo et al. 2018), suggest that the appearance of a second melting peak in plasticized PHB samples could be attributed to the degradation of the polymer and the presence of different molecular weight samples. The higher is the OLA ratio inside the polymer, the wider is the melting band and Δ*H*_m_ decreases. This may be related with the increased mobility inside the polymer since the crystalline cores are dispersed and consequently, showing nonuniform crystal morphology. Furthermore, the enthalpy is reduced due to the absence of a melting peak in OLA (Meereboer et al. 2020).

**Table 3:** DSC second heating scan data for the different PHBV-OLA samples

Sample	*T*_m_ (°C)	Δ*H*_m_ (J/g)	*X*_c_ (%)
PHBV-v	169.7	100.3	69
PHBV-10OLA-v	166.0	93.1	71
PHBV-15OLA-v	163.4	86.2	69
PHBV-20OLA-v	159.1	79.6	68
PHBV-r	168.9	102.8	70
PHBV-10OLA-r	166.2	95.6	73
PHBV-15OLA-r	163.8	90.8	73
PHBV-20OLA-r	161.3	85.0	73

Another important difference between the DSC curves is the decrease of the melting temperature with the amount of OLA. This behavior is in good agreement with the plasticizing effect of OLA, since the depression of the melting point could be related to the reduced interactions between the PHBV chains, because of the dispersion of the plasticizer molecules within the polymer matrix. Similar results in PHBV samples were observed by Barbosa et al. (Barbosa et al. 2021) with an oligomeric polyester-based plasticizer; Martino et al. (Martino et al. 2015) using ATBC, PEG, and GTA; Requena et al. (Requena et al. 2016) using PEG and lauric and stearic acid; and by García-García et al. (Garcia-Garcia et al. 2016) in PHB plasticized with modified vegetable oils.

This decrease of the melting temperature could improve the processability of PHBV, because PHB polymers and copolymers have a narrow processing window. Finally, the second heating results show that the accelerated ageing and the mechanical recycling do not have an important effect on the thermal behavior of the samples, despite the decrease of the molecular weight of the polymer. Similar results were obtained by Zaverl et al. (Zaverl et al. 2012) after subjecting a PHBV to up to two reprocessing cycles and by Dedieu et al. (Dedieu et al. 2022a) in a PHBV reprocessed four times.

**Fig. 8:**
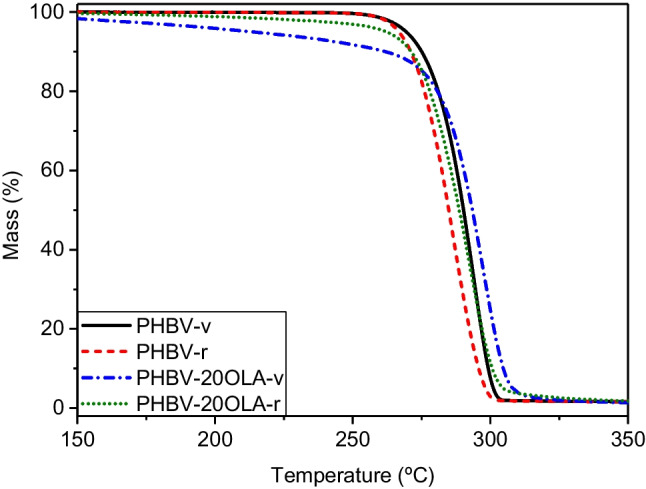
TGA curves of PHBV and PHBV-20OLA formulations before and after recycling

Thermal stability is another important property, especially from a processability point of view. The effect of OLA and mechanical recycling on the thermal stability of PHBV was studied by means of TGA tests, and the results are summarized in Fig. 8 and Table 4. The OLA additive showed an onset decomposition temperature of 185 °C, in good agreement with the data available in the literature (Cicogna et al. 2017). Regarding the PHBV, it shows a one-stage decomposition process, corresponding to a random chain scission by β-elimination mechanism (Grassie et al. 1984). The addition of OLA to PHBV led to a decrease of up to 14 °C in the thermal stability of PHBV, due to the low thermal stability of OLA. Similar results were obtained by Barbosa et al. (Barbosa et al. 2021) in PHBV plasticized with an oligomeric polyester. Burgos et al. (Burgos et al. 2013) also reported an important decrease of the thermal stability of PLA plasticized with OLA. In the same line are the results reported by Requena et al. (Requena et al. 2016), which observed a decrease on the thermal stability of PHBV plasticized with PEG, lauric acid, and stearic acid. Wang et al. (Wang et al. 2008) also reported a decrease in the onset decomposition temperature of PHB plasticized with ATBC.

**Table 4:** TGA results for the different PHBV-OLA materials

Sample	*T*_10_ (°C)	*T*_max_ (°C)
Pure OLA	185.9	311.9
PHBV-v	275.1	293.5
PHBV-10OLA-v	276.1	297.5
PHBV-15OLA-v	272.9	298.7
PHBV-20OLA-v	261.9	297.4
PHBV-r	271.0	286.4
PHBV-10OLA-r	278.1	297.5
PHBV-15OLA-r	260.8	277.7
PHBV-20OLA-r	271.2	292.4

Regarding the effect of mechanical recycling on the PHBV-OLA formulations, the behavior is more complex. On the one hand, in the pure PHBV sample, a small decrease on the thermal stability was observed. This result could be explained by the slight reduction of the molecular weight during recycling, as it has been pointed out in previous studies in and PHBV subjected to several reprocessing cycles (Dedieu et al. 2022a, Zaverl et al. 2012). On the other hand, the plasticized samples show an overall increase in the thermal stability. However, the intrinsic viscosity values indicate that the samples with OLA also show a decrease in the molecular weight. Therefore, the observed increase in the thermal stability could be explained by the elimination of the plasticizer during recycling, since OLA might be responsible of the decrease in the thermal stability of PHBV. It can be seen on Table 4 that thermal decomposition of OLA starts around 185 °C, which is within the range of melt processing temperatures used in this work. Furthermore, the plasticizer might have suffered some degradation during the first melt processing and accelerated ageing, generating reactive terminal groups that promote the thermal decomposition of OLA (Burgos et al. 2014), thus increasing the decomposition of the plasticizer during the second processing step. Therefore, it is possible that the OLA plasticizer partially decomposed during the second melt processing, thus explaining the increase on the thermal stability of the material.

### Mechanical properties

Mechanical properties, such as tensile modulus or tensile strength, are one of the key aspects in packaging applications. The effect of the addition of OLA and mechanical recycling on the Young modulus (*E*), tensile strength (*σ*_max_), and elongation ant break (*ε*_max_) of PHBV was analyzed by means of tensile tests. The results are summarized in Table 5. Firstly, in the virgin samples, the addition of OLA leads to a notorious decrease of the tensile modulus (58% decrease in the sample loaded with 20%wt. OLA), a decrease of the tensile strength, and an increase of the maximum elongation (from 1.6% in PHBV-v to 4.3% in PHBV-15OLA-v). This behavior suggests that OLA has a plasticizing effect in PHBV. The relatively small OLA molecules intercalate between the PHBV chains, increasing the free volume and the mobility of the molecular segments, which leads to increased flexibility, although the tensile strength of the materials is reduced. Similar results were observed in previous studies of PHBV and PHB plasticized with PEG, ATBC, epoxidized soybean oil, and tributyrin (TBL) (D'Amico et al. 2016; Garcia-Garcia et al. 2016; Jost and Langowski 2015; Panaitescu et al. 2017; Requena et al. 2016). It is worth to note that the addition of 20%wt. OLA led to a slight decrease of the elongation at break. This result suggests a phase separation, which results in mechanically fragile amorphous region that facilitates crack propagation (Barbosa et al. 2021).

**Table 5:** Mechanical properties of the PHBV-OLA samples

Sample	*E* (MPa)	*σ*_max_ (MPa)	*ε*_max_ (%)
PHBV-v	2170 ± 70	24.9 ± 2.5	1.6 ± 0.3
PHBV-10OLA-v	1410 ± 60	20.0 ± 1.4	3.0 ± 0.1
PHBV-15OLA-v	1150 ± 40	19.5 ± 1.6	4.3 ± 0.5
PHBV-20OLA-v	900 ± 50	14.3 ± 1.5	3.4 ± 0.4
PHBV-r	2140 ± 70	24.4 ± 1.5	1.6 ± 0.3
PHBV-10OLA-r	1880 ± 80	23.2 ± 0.8	1.8 ± 0.2
PHBV-15OLA-r	1520 ± 150	20.0 ± 1.1	2.0 ± 0.2
PHBV-20OLA-r	1220 ± 60	14.3 ± 1.6	1.9 ± 0.2

Regarding the effect of mechanical recycling, Table 5 shows that the recycled plasticized materials show higher modulus and lower elongation at break than their virgin counterparts. This result is in good agreement with those observed by means of TGA and FTIR, which suggest that mechanical recycling led to the partial elimination of OLA.

## Conclusions

The effect of mechanical recycling on the structure and properties of PHBV and PHBV-OLA formulations was studied. A commercial PHBV with 1.6% HV was compounded with 10, 15, and 20%wt. OLA as a plasticizer, subjected to an accelerated ageing protocol, followed by a demanding washing process and reprocessed by extrusion and compression molding. On the one hand, the ageing and mechanical recycling processes led only to a slight degradation in the pure PHBV material, accompanied by small changes in the thermal stability and mechanical properties of the polymer. On the other hand, the PHBV-OLA formulations showed a more severe degradation, probably due to the terminal groups of the oligomer acting as a catalyst of the thermomechanical degradation of the PHBV matrix.

Thermal and mechanical characterization showed that OLA has a plasticizing effect on PHBV, leading to lower melting temperatures, lower Young modulus, and higher elongation at break values. However, part of the OLA is eliminated during the mechanical recycling process, resulting in recycled PHBV formulations with lower ductility, increased stiffness, and increased thermal stability. These results point out the plasticizing effect of OLA, favoring the processability and increasing the flexibility and ductility of PHBV. However, especial attention should be paid to their behavior during mechanical recycling since recycled materials with poor performance could be obtained.

Overall, the results suggest that mechanical recycling could be an interesting alternative for the valorization of PHBV wastes, which would translate in a reduction of the raw materials, emissions, and energy associated to the production of the virgin plastics. Furthermore, it must be considered that, although PHBV is a compostable polymer, the plastics based on PHBV can include different additives that complicate the correct management of the wastes. The possibility of mechanically recycle PHBV could open new applications and markets for the residues of these plastics, thus contributing to reduce the issues associated to the waste management. The results obtained in this work also reveal that the presence of additives such as plasticizers could lead to further degradation and significant changes in the performance of the recycled materials, thus needing special attention.

## Data Availability

Data is available upon reasonable request to the corresponding author.
